# Poly[(μ_3_-nicotinato-κ^3^ *O*:*O*′:*N*)(μ_2_-nicotinato-κ^3^ *O*,*O*′:*N*)iron(II)]

**DOI:** 10.1107/S1600536808011045

**Published:** 2008-04-26

**Authors:** Seik Weng Ng

**Affiliations:** a Department of Chemistry, University of Malaya, 50603 Kuala Lumpur, Malaysia

## Abstract

In the crystal structure of the title compound, [Fe(C_6_H_4_NO_2_)_2_]_*n*_, one nicotinate group *O*,*O*′-chelates one Fe atom and binds through the N atom to the other Fe atom; the second nicotinate group bridges three Fe atoms through the N and two O atoms. The μ_2_- and μ_3_-bridging modes of the two nicotinate groups result in a polymeric three-dimensional network structure. The Fe atom shows octa­hedral coordination geometry but one of the Fe—O bonds is somewhat long [2.522 (2) Å].

## Related literature

For zwitterionic tetra­aquadi(nicotinato-*κN*)iron(II), see: Liang *et al.* (2005 ▶).
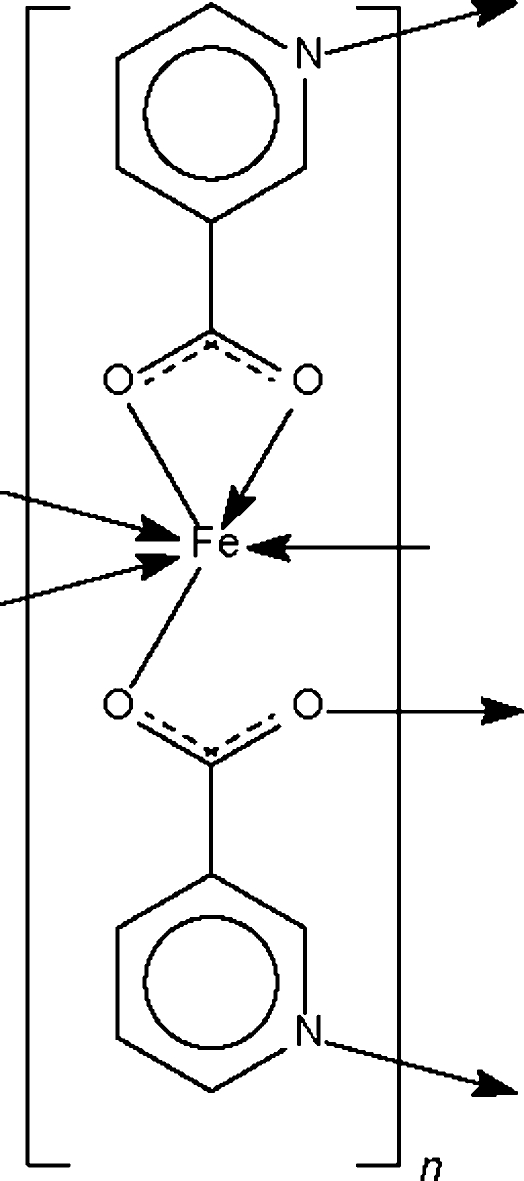


## Experimental

### 

#### Crystal data

[Fe(C_6_H_4_NO_2_)_2_]
*M* *_r_* = 300.05
Monoclinic, 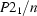

*a* = 10.8771 (7) Å
*b* = 9.6066 (6) Å
*c* = 12.7284 (8) Å
β = 111.619 (1)°
*V* = 1236.5 (1) Å^3^
*Z* = 4
Mo *K*α radiation
μ = 1.23 mm^−1^
*T* = 295 (2) K
0.41 × 0.34 × 0.25 mm

#### Data collection

Bruker SMART APEX diffractometer
Absorption correction: multi-scan (*SADABS*; Sheldrick, 1996 ▶) *T*_min_ = 0.564, *T*_max_ = 0.749
7255 measured reflections
2762 independent reflections
2428 reflections with *I* > 2σ(*I*)
*R*_int_ = 0.018

#### Refinement

*R*[*F* ^2^ > 2σ(*F* ^2^)] = 0.028
*wR*(*F* ^2^) = 0.078
*S* = 1.02
2762 reflections
172 parameters
H-atom parameters constrained
Δρ_max_ = 0.24 e Å^−3^
Δρ_min_ = −0.27 e Å^−3^

### 

Data collection: *SMART* (Bruker, 2004 ▶); cell refinement: *SAINT* (Bruker, 2004 ▶); data reduction: *SAINT*; program(s) used to solve structure: *SHELXS97* (Sheldrick, 2008 ▶); program(s) used to refine structure: *SHELXL97* (Sheldrick, 2008 ▶); molecular graphics: *X-SEED* (Barbour, 2001 ▶) and *OLEX* (Dolomanov *et al.*, 2003 ▶); software used to prepare material for publication: *publCIF* (Westrip, 2008 ▶).

## Supplementary Material

Crystal structure: contains datablocks global, I. DOI: 10.1107/S1600536808011045/xu2410sup1.cif

Structure factors: contains datablocks I. DOI: 10.1107/S1600536808011045/xu2410Isup2.hkl

Additional supplementary materials:  crystallographic information; 3D view; checkCIF report

## Figures and Tables

**Table:** 

Fe1—O1	2.522 (2)
Fe1—O2	2.072 (1)
Fe1—O3	2.012 (1)
Fe1—O4^i^	2.061 (1)
Fe1—N1^ii^	2.212 (1)
Fe1—N2^iii^	2.224 (1)

**Table:** 

O1—Fe1—O2	56.18 (5)
O1—Fe1—O3	96.95 (5)
O1—Fe1—O4^i^	142.30 (5)
O1—Fe1—N1^ii^	89.36 (5)
O1—Fe1—N2^iii^	93.18 (5)
O2—Fe1—O3	153.10 (6)
O2—Fe1—O4^i^	86.23 (5)
O2—Fe1—N1^ii^	92.17 (5)
O2—Fe1—N2^iii^	90.93 (5)
O3—Fe1—O4^i^	120.67 (6)
O3—Fe1—N1^ii^	88.50 (5)
O3—Fe1—N2^iii^	89.17 (5)
O4^i^—Fe1—N1^ii^	89.39 (6)
O4^i^—Fe1—N2^iii^	89.83 (6)
N1^ii^—Fe1—N2^iii^	176.74 (5)

Symmetry codes: (i) 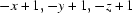; (ii) 
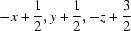
; (iii) 
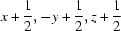
.

**Fig. 1.**
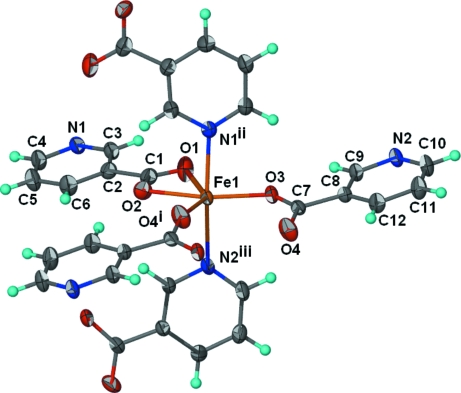
50% Probability thermal ellipsoid plot illustrating the octahedral geometry at iron.

**Fig. 2.**
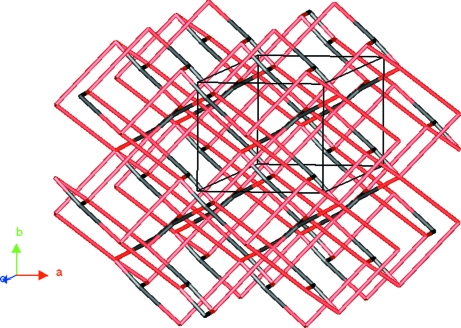
OLEX (Dolomanov et al., 2003) illustration of the three-dimensional network motif.

## supplementary crystallographic information

### Comment

The crystal structures of a large number of divalent metal dinicotinates are known; the compounds exists as water-coordinated compounds in which the nicotinate ion binds through the aromatic N atom and not through the carboxyl group, as exemplified by tetraaquadinicotinatoiron(II). The report on this compound lists the crystal structures of tetraaquametal dinicotinates (Liang *et al.*, 2005). Tetraaquadinicotinatoiron is synthesized by reaction of the metal salt with nicotinic acid under aqueous conditions; under hydrothermal conditions, the synthesis has yielded the anhydrous compound (I). Iron dinicotinate (Fig. 1) has the nicotinate group engaged into two types of bridging interactions; one group *O*,*O*'-chelate to one Fe atom and binds through the N atom to the other Fe atom; the second nicotinate group bridges three Fe atoms through the N and two O atoms. The µ_2_ and µ_3_ bridging modes of the two nicotinate groups result in a polymeric three-dimensional network structure (Fig. 2). The Fe atom shows the common octahedral coordination geometry but one of the Fe–O bonds is somewhat long (Table 1).

### Experimental

Iron powder (0.056 g, 1 mmol), nicotinic acid (0.218 g 2 mmol) and water (10 ml) heated in a 23-ml, Teflon-lined, Parr bomb at 423 K for 3 days. The bomb was cooled to room temperature at a rate of 10 K per min to give yellow block-shaped crystals (in 10% yield based on nicotinic acid rate of 10 *^o^*C.h-1. The yellow block crystals of iron dinicoinate were obtained (yield 8.2% based on nicotinic acid).

### Refinement

Carbon-bound H-atoms were placed in calculated positions (C—H 0.93 Å) and were included in the refinement in the riding model approximation, with *U*(H) set to 1.2*U*(C).

### Crystal data

**Table:**

[Fe(C_6_H_4_NO_2_)_2_]	*F*_000_ = 608
*M**_r_* = 300.05	*D*_x_ = 1.612 Mg m^−^^3^
Monoclinic, *P*2_1_/*n*	Mo *K*α radiation λ = 0.71073 Å
Hall symbol: -P 2yn	Cell parameters from 6064 reflections
*a* = 10.8771 (7) Å	θ = 2.1–27.5º
*b* = 9.6066 (6) Å	µ = 1.23 mm^−^^1^
*c* = 12.7284 (8) Å	*T* = 295 (2) K
β = 111.619 (1)º	Block, yellow
*V* = 1236.5 (1) Å^3^	0.41 × 0.34 × 0.25 mm
*Z* = 4	

### Data collection

**Table:**

Bruker APEX diffractometer	2762 independent reflections
Radiation source: fine-focus sealed tube	2428 reflections with *I* > 2σ(*I*)
Monochromator: graphite	*R*_int_ = 0.018
*T* = 295(2) K	θ_max_ = 27.5º
φ and ω scans	θ_min_ = 2.1º
Absorption correction: multi-scan(SADABS; Sheldrick, 1996)	*h* = −14→14
*T*_min_ = 0.564, *T*_max_ = 0.749	*k* = −10→12
7255 measured reflections	*l* = −13→16

### Refinement

**Table:**

Refinement on *F*^2^	Secondary atom site location: difference Fourier map
Least-squares matrix: full	Hydrogen site location: inferred from neighbouring sites
*R*[*F*^2^ > 2σ(*F*^2^)] = 0.028	H-atom parameters constrained
*wR*(*F*^2^) = 0.078	*w* = 1/[σ^2^(*F*_o_^2^) + (0.0475*P*)^2^ + 0.2265*P*] where *P* = (*F*_o_^2^ + 2*F*_c_^2^)/3
*S* = 1.02	(Δ/σ)_max_ = 0.001
2762 reflections	Δρ_max_ = 0.24 e Å^−^^3^
172 parameters	Δρ_min_ = −0.27 e Å^−^^3^
Primary atom site location: structure-invariant direct methods	Extinction correction: none

### Fractional atomic coordinates and isotropic or equivalent isotropic displacement parameters (Å^2^)

**Table:**

	*x*	*y*	*z*	*U*_iso_*/*U*_eq_	
Fe1	0.40603 (2)	0.37707 (2)	0.580119 (18)	0.02707 (10)	
O1	0.28044 (15)	0.19952 (16)	0.64714 (12)	0.0540 (4)	
O2	0.43525 (13)	0.34442 (14)	0.74858 (11)	0.0398 (3)	
O3	0.32118 (11)	0.33429 (14)	0.41409 (10)	0.0345 (3)	
O4	0.45693 (13)	0.46511 (14)	0.36328 (12)	0.0477 (3)	
N1	0.25364 (13)	0.02956 (15)	0.94060 (12)	0.0335 (3)	
N2	0.06748 (13)	0.27149 (15)	0.09200 (12)	0.0335 (3)	
C1	0.35420 (18)	0.24585 (18)	0.73955 (15)	0.0371 (4)	
C2	0.35386 (17)	0.18565 (18)	0.84835 (14)	0.0336 (4)	
C3	0.25803 (17)	0.09027 (18)	0.84724 (15)	0.0344 (4)	
H3	0.1932	0.0671	0.7780	0.041*	
C4	0.34759 (18)	0.0657 (2)	1.03903 (15)	0.0396 (4)	
H4	0.3462	0.0246	1.1047	0.048*	
C5	0.4460 (2)	0.1601 (2)	1.04838 (16)	0.0450 (5)	
H5	0.5090	0.1824	1.1187	0.054*	
C6	0.44980 (18)	0.2211 (2)	0.95150 (16)	0.0415 (4)	
H6	0.5156	0.2849	0.9554	0.050*	
C7	0.35595 (15)	0.39427 (16)	0.34127 (14)	0.0283 (3)	
C8	0.26579 (15)	0.37708 (15)	0.22048 (14)	0.0281 (3)	
C9	0.15745 (16)	0.29047 (17)	0.19585 (13)	0.0314 (3)	
H9	0.1464	0.2424	0.2552	0.038*	
C10	0.08654 (19)	0.3417 (2)	0.00799 (15)	0.0400 (4)	
H10	0.0251	0.3306	−0.0651	0.048*	
C11	0.19213 (19)	0.4291 (2)	0.02432 (15)	0.0432 (4)	
H11	0.2013	0.4753	−0.0366	0.052*	
C12	0.28458 (18)	0.44749 (18)	0.13239 (15)	0.0368 (4)	
H12	0.3572	0.5054	0.1456	0.044*	

### Atomic displacement parameters (Å^2^)

**Table:**

	*U*^11^	*U*^22^	*U*^33^	*U*^12^	*U*^13^	*U*^23^
Fe1	0.02436 (14)	0.03310 (15)	0.01968 (14)	−0.00138 (8)	0.00333 (10)	−0.00096 (8)
O1	0.0695 (10)	0.0593 (9)	0.0300 (7)	−0.0046 (8)	0.0145 (7)	0.0019 (6)
O2	0.0405 (7)	0.0448 (7)	0.0379 (7)	0.0017 (6)	0.0188 (6)	0.0113 (5)
O3	0.0304 (6)	0.0479 (7)	0.0207 (6)	−0.0014 (5)	0.0042 (5)	−0.0011 (5)
O4	0.0393 (7)	0.0507 (8)	0.0417 (8)	−0.0193 (6)	0.0014 (6)	−0.0023 (6)
N1	0.0301 (7)	0.0397 (8)	0.0282 (7)	−0.0009 (6)	0.0080 (6)	0.0040 (6)
N2	0.0311 (7)	0.0408 (8)	0.0225 (7)	−0.0059 (6)	0.0027 (6)	0.0003 (6)
C1	0.0419 (9)	0.0396 (9)	0.0332 (9)	0.0092 (8)	0.0176 (8)	0.0055 (7)
C2	0.0358 (9)	0.0359 (9)	0.0311 (9)	0.0018 (7)	0.0146 (7)	0.0027 (7)
C3	0.0331 (8)	0.0396 (9)	0.0267 (8)	0.0007 (7)	0.0065 (7)	0.0021 (7)
C4	0.0389 (9)	0.0504 (11)	0.0267 (9)	−0.0055 (8)	0.0089 (7)	0.0053 (8)
C5	0.0426 (10)	0.0566 (12)	0.0285 (9)	−0.0122 (9)	0.0045 (8)	0.0005 (8)
C6	0.0403 (9)	0.0462 (10)	0.0368 (10)	−0.0110 (8)	0.0127 (8)	0.0018 (8)
C7	0.0255 (8)	0.0286 (8)	0.0262 (8)	0.0026 (6)	0.0044 (6)	−0.0032 (6)
C8	0.0270 (8)	0.0311 (8)	0.0240 (8)	−0.0006 (6)	0.0066 (6)	−0.0023 (6)
C9	0.0305 (8)	0.0383 (9)	0.0224 (8)	−0.0044 (7)	0.0061 (6)	0.0015 (6)
C10	0.0433 (10)	0.0458 (10)	0.0222 (8)	−0.0046 (8)	0.0017 (7)	0.0014 (7)
C11	0.0519 (11)	0.0481 (10)	0.0274 (9)	−0.0099 (9)	0.0121 (8)	0.0059 (8)
C12	0.0378 (9)	0.0398 (9)	0.0313 (9)	−0.0093 (7)	0.0109 (7)	0.0003 (7)

### Geometric parameters (Å, °)

**Table:**

Fe1—O1	2.522 (2)	C2—C3	1.384 (2)
Fe1—O2	2.072 (1)	C2—C6	1.385 (3)
Fe1—O3	2.012 (1)	C3—H3	0.9300
Fe1—O4^i^	2.061 (1)	C4—C5	1.375 (3)
Fe1—N1^ii^	2.212 (1)	C4—H4	0.9300
Fe1—N2^iii^	2.224 (1)	C5—C6	1.379 (3)
O1—C1	1.237 (2)	C5—H5	0.9300
O2—C1	1.270 (2)	C6—H6	0.9300
O3—C7	1.262 (2)	C7—C8	1.497 (2)
O4—C7	1.233 (2)	C8—C9	1.381 (2)
O4—Fe1^i^	2.0611 (12)	C8—C12	1.388 (2)
N1—C4	1.338 (2)	C9—H9	0.9300
N1—C3	1.340 (2)	C10—C11	1.375 (3)
N1—Fe1^iv^	2.2124 (14)	C10—H10	0.9300
N2—C9	1.336 (2)	C11—C12	1.384 (2)
N2—C10	1.343 (2)	C11—H11	0.9300
N2—Fe1^v^	2.2243 (14)	C12—H12	0.9300
C1—C2	1.502 (2)		
			
O1—Fe1—O2	56.18 (5)	N1—C3—C2	123.49 (16)
O1—Fe1—O3	96.95 (5)	N1—C3—H3	118.3
O1—Fe1—O4^i^	142.30 (5)	C2—C3—H3	118.3
O1—Fe1—N1^ii^	89.36 (5)	N1—C4—C5	123.64 (17)
O1—Fe1—N2^iii^	93.18 (5)	N1—C4—H4	118.2
O2—Fe1—O3	153.10 (6)	C5—C4—H4	118.2
O2—Fe1—O4^i^	86.23 (5)	C4—C5—C6	118.78 (18)
O2—Fe1—N1^ii^	92.17 (5)	C4—C5—H5	120.6
O2—Fe1—N2^iii^	90.93 (5)	C6—C5—H5	120.6
O3—Fe1—O4^i^	120.67 (6)	C5—C6—C2	118.87 (17)
O3—Fe1—N1^ii^	88.50 (5)	C5—C6—H6	120.6
O3—Fe1—N2^iii^	89.17 (5)	C2—C6—H6	120.6
O4^i^—Fe1—N1^ii^	89.39 (6)	O4—C7—O3	124.60 (16)
O4^i^—Fe1—N2^iii^	89.83 (6)	O4—C7—C8	119.06 (15)
N1^ii^—Fe1—N2^iii^	176.74 (5)	O3—C7—C8	116.33 (14)
C1—O1—Fe1	80.56 (11)	C9—C8—C12	118.53 (15)
C1—O2—Fe1	100.52 (11)	C9—C8—C7	118.75 (15)
C7—O3—Fe1	122.17 (11)	C12—C8—C7	122.70 (15)
C7—O4—Fe1^i^	162.57 (13)	N2—C9—C8	124.03 (15)
C4—N1—C3	116.93 (15)	N2—C9—H9	118.0
C4—N1—Fe1^iv^	125.34 (12)	C8—C9—H9	118.0
C3—N1—Fe1^iv^	117.73 (11)	N2—C10—C11	123.46 (16)
C9—N2—C10	116.58 (14)	N2—C10—H10	118.3
C9—N2—Fe1^v^	115.27 (11)	C11—C10—H10	118.3
C10—N2—Fe1^v^	128.13 (12)	C10—C11—C12	119.27 (17)
O1—C1—O2	122.67 (17)	C10—C11—H11	120.4
O1—C1—C2	121.14 (17)	C12—C11—H11	120.4
O2—C1—C2	116.18 (16)	C11—C12—C8	118.12 (16)
C3—C2—C6	118.28 (16)	C11—C12—H12	120.9
C3—C2—C1	120.21 (16)	C8—C12—H12	120.9
C6—C2—C1	121.49 (16)		
			
O3—Fe1—O1—C1	177.00 (11)	C1—C2—C3—N1	178.18 (15)
O4^i^—Fe1—O1—C1	−6.40 (16)	C3—N1—C4—C5	0.0 (3)
O2—Fe1—O1—C1	−1.54 (10)	Fe1^iv^—N1—C4—C5	179.39 (16)
N1^ii^—Fe1—O1—C1	−94.59 (11)	N1—C4—C5—C6	−0.4 (3)
N2^iii^—Fe1—O1—C1	87.45 (11)	C4—C5—C6—C2	0.4 (3)
O3—Fe1—O2—C1	−1.69 (18)	C3—C2—C6—C5	0.1 (3)
O4^i^—Fe1—O2—C1	178.53 (11)	C1—C2—C6—C5	−178.59 (17)
N1^ii^—Fe1—O2—C1	89.28 (11)	Fe1^i^—O4—C7—O3	−68.9 (5)
N2^iii^—Fe1—O2—C1	−91.70 (11)	Fe1^i^—O4—C7—C8	110.7 (4)
O1—Fe1—O2—C1	1.51 (10)	Fe1—O3—C7—O4	12.9 (2)
O4^i^—Fe1—O3—C7	5.56 (14)	Fe1—O3—C7—C8	−166.66 (10)
O2—Fe1—O3—C7	−174.18 (11)	O4—C7—C8—C9	175.90 (15)
N1^ii^—Fe1—O3—C7	93.97 (13)	O3—C7—C8—C9	−4.5 (2)
N2^iii^—Fe1—O3—C7	−83.75 (13)	O4—C7—C8—C12	−5.9 (2)
O1—Fe1—O3—C7	−176.85 (12)	O3—C7—C8—C12	173.73 (15)
Fe1—O1—C1—O2	2.49 (16)	C10—N2—C9—C8	0.4 (3)
Fe1—O1—C1—C2	−176.46 (16)	Fe1^v^—N2—C9—C8	179.22 (13)
Fe1—O2—C1—O1	−3.0 (2)	C12—C8—C9—N2	−1.1 (3)
Fe1—O2—C1—C2	175.95 (12)	C7—C8—C9—N2	177.20 (15)
O1—C1—C2—C3	−9.1 (3)	C9—N2—C10—C11	0.3 (3)
O2—C1—C2—C3	171.91 (16)	Fe1^v^—N2—C10—C11	−178.38 (15)
O1—C1—C2—C6	169.55 (18)	N2—C10—C11—C12	−0.2 (3)
O2—C1—C2—C6	−9.5 (2)	C10—C11—C12—C8	−0.5 (3)
C4—N1—C3—C2	0.4 (3)	C9—C8—C12—C11	1.1 (3)
Fe1^iv^—N1—C3—C2	−178.97 (13)	C7—C8—C12—C11	−177.12 (16)
C6—C2—C3—N1	−0.5 (3)		

Symmetry codes: (i) −*x*+1, −*y*+1, −*z*+1; (ii) −*x*+1/2, *y*+1/2, −*z*+3/2; (iii) *x*+1/2, −*y*+1/2, *z*+1/2; (iv) −*x*+1/2, *y*−1/2, −*z*+3/2; (v) *x*−1/2, −*y*+1/2, *z*−1/2.
